# Prognostic impact of circulating tumor cells in patients with ampullary cancer

**DOI:** 10.1002/jcp.26353

**Published:** 2018-01-04

**Authors:** Bo Sun, Han Liu, Shengnan Wang, Jianbin Xiang, Xingdang Liu

**Affiliations:** ^1^ Department of General Surgery Huashan Hospital Fudan University Shanghai China; ^2^ Department of Nuclear Medicine Huashan Hospital Fudan University Shanghai China

**Keywords:** ampullary cancer, circulating tumor cells, prognostic factors

## Abstract

Circulating tumor cells (CTCs) are an important topic of investigation for both basic and clinical cancer research. In this prospective study, we evaluated the clinical role of CTCs in ampullary cancer. We analyzed blood samples from 62 consecutively diagnosed patients with ampullary adenocarcinoma and 24 healthy controls for their CTC content. Combined data from immunostaining of CD45, 4′,6‐diamidino‐2‐phenylindole (DAPI), and fluorescence in situ hybridization with a chromosome 8 centromere (CEP8) probe were used to identify CTCs; cells that were CD45‐/DAPI+/CEP8>2 were considered CTCs. The Cox proportional hazards model was used to assess the relationship between CTCs, clinical characteristics, and patient outcomes. We detected ≥2 CTCs/3.2 ml whole blood in 43 of 62 patients (69.4%), as well as ≥5 CTCs/3.2 ml in 16 of these patients (25.8%). A CTC cutoff value of 2 cells/3.2 ml achieved 69.4% sensitivity and 95.8% specificity as a diagnostic tool; CTCs were associated with tumor burden. CTC levels ≥3/3.2 ml (hazard ratio [HR]: 2.5, 95% confidence interval [CI]: (1.2–5.2), *p* = 0.014) and ≥5/3.2 ml (HR: 3.5, 95% CI: 1.7–7.3, *p *< 0.001) were both associated with shorter disease‐free survival. Moreover, ≥3 CTCs/3.2 ml (HR: 2.7, 95% CI: 1.2–6.3, *p* = 0.019) and ≥5 CTCs/3.2 ml (HR: 3.8, 95% CI: 1.8–8.5, *p < *0.001) were predictive of shorter overall survival. CTC assessment may help identify patients with ampullary cancer who are at high risk of an unfavorable outcome.

**Abbreviations**
AJCC: American Joint Committee on Cancer
CEA: carcinoembryonic antigen
CEP8: chromosome 8 centromere
CI: confidence interval
CTC: circulating tumor cell
DAPI: 4',6‐diamidino‐2‐phenylindole
DFS: disease‐free survival
EpCAM: epithelial cell adhesion molecules
FISH: fluorescence in situ hybridization
HCC: hepatocellular carcinoma
HR: hazard ratio
ISET: isolation by size of epithelial tumor cells
OS: overall survival
PCR: polymerase chain reaction
PFS: progression‐free survival
ROC: receiver operating characteristics
SSC: saline‐sodium citrate
UICC: Union for International Cancer Control

## 1 BACKGROUND

Ampullary cancers are rare malignant neoplasms that arise from the ampulla of Vater (Albores‐Saavedra, Schwartz, Batich, & Henson, 2009). Although this disease represents only 0.5% of gastrointestinal cancers and 7% of cancers arising in the region of the head of the pancreas, the rates of ampullary cancer have been rising annually over the past few years (Ang, Shia, Tang, Katabi, & Klimstra, 2014; Siegel, Miller, & Jemal, 2017). Despite major efforts in recent decades, patients with ampullary cancer continue to experience poor prognosis and a high death rate. It is imperative to identify the potential biological markers for early diagnosis, novel therapeutic strategies, and prognostic predictors in patients with ampullary cancer.

Circulating tumor cells (CTCs) are minute numbers of tumor cells that escape from primary tumors and metastatic foci and enter the bloodstream (Cristofanilli, 2006). CTCs have been discovered in most major cancers, although at varying rates (Allard et al., 2004). Clinical studies have suggested that CTCs are correlated with poor progression‐free survival (PFS) and overall survival (OS) in patients with breast cancer, colon cancer, and cholangiocarcinoma, as well as other malignancies (Cohen et al., 2008; Rack et al., 2014; Sonpavde & Antonarakis, 2017; Yang et al., 2016). These cells have also been utilized for guiding clinical management, evaluating curative efficacy, and monitoring tumor recurrence (Plaks, Koopman, & Werb, 2013). However, the clinical significance of CTCs in ampullary cancer has not been explored as thoroughly as it has for other epithelial cancers.

As CTCs are scarce in the blood stream, a number of novel methodologies have been developed to improve the efficiency and precision of their detection. The CellSearch system is the only CTC detection technology approved by the Food and Drug Administration (Allard et al., 2004); the procedure relies on cytokeratin staining of cells in blood samples. However, the CellSearch system is limited in that epithelial cell adhesion molecules (EpCAMs) and cytokeratin are not expressed in all CTCs (Nagrath, Jack, Sahai, & Simeone, 2016). Aneuploidy, a common manifestation of chromosome instability, is a hallmark of malignant solid tumors (Danielsen, Pradhan, & Novelli, 2016). As chromosome numbers are reflected by the chromosome 8 centromere (CEP8) that can be detected using fluorescence in situ hybridization (FISH), aneuploidy detection in peripheral blood provides a fresh avenue for CTC detection (Pecot et al., 2011). The feasibility of this approach has been demonstrated since the vast majority of circulating epithelial cells have been found to be aneuploid cells derived from primary tumors (Fehm et al., 2002). This method shows improved isolation efficiency in solid tumors compared to conventional cytokeratin‐based methods (Ning et al., 2014).

In this study, we detected and characterized CTCs in 86 blood samples from 62 consecutive patients diagnosed with ampullary adenocarcinoma, as well as from 24 healthy control subjects using a method that combines immunostaining of CD45, 4′,6‐diamidino‐2‐phenylindole (DAPI), and CEP8‐FISH. We further explored the relationships between CTCs and clinical parameters and outcomes.

## 2 METHODS

### 2.1 Patients and clinical information

This prospective study was restricted to consecutively diagnosed ampullary adenocarcinoma patients treated at the Fudan University‐Affiliated Huashan Hospital. Sixty‐two patients with pathologically proven ampullary adenocarcinoma and 24 healthy controls were enrolled between March 2012 and December 2015. Written informed consent was obtained from all subjects. Pathological and radiological examination provided evidence for the diagnosis of ampullary cancer. Data on clinical variables including sex, age, tumor grade, tumor size, lymph node invasion, distant metastases, and selected laboratory results were gathered. Ampullary cancer TNM staging was performed according to the American Joint Committee on Cancer (AJCC) 2010 criteria (Edge & Compton, 2010). Treatments that patients received after enrollment were reviewed, and patients were followed until February 28, 2017. DFS was defined as the interval between the initial surgery and documented manifestation of recurrent disease (Eisenhauer et al., 2009). OS was calculated from the date of initial surgery to the date of death or last contact. Follow‐up data were available for all included patients. This study was conducted with the approval of the Ethics Committee of Fudan University‐Affiliated Huashan Hospital in adherence with the principles of the Declaration of Helsinki.

### 2.2 Sample collection and polyploidy detection

Whole peripheral blood samples were obtained via venipuncture from healthy subjects and from patients with ampullary cancer before their undergoing invasive tests and therapeutic measures. Tubes containing acid citrate dextrose‐anticoagulant (Becton Dickinson, NJ) were used to obtain 3.2 ml whole blood samples for CTC detection. All blood samples were processed within 48 hr of collection.

Strategies for CTC isolation and detection were similar to those described previously (Ning et al., 2014). In brief, 4 ml of collected blood (including 0.8 ml of anticoagulant) was centrifuged to separate cells from whole blood. Next, red blood cells were dissolved using hypotonic hemolysis, and residual cell particles were resuspended in phosphate buffer solution. CTCs were cultured with anti‐CD45 monoclonal antibody‐coated magnetic beads (Life Technologies, Carlsbad, CA). Subsequently, the magnetic beads (which were mostly bound to leukocytes) were separated from the cell suspension by a magnetic stand (Promega, Madison, WI), and the leukocytes were smeared onto slides and fixed for identification. Specimens were dipped in 2× saline‐sodium citrate (SSC) buffer at 37 °C for 15 min, then dehydrated by sequential exposure to 75%, 85%, and 100% ethanol for 3 min each. Spectrum Orange‐labeled CEP8 was added (Abbott Molecular Diagnostics, Des Plaines, IL). Specimens were denatured at 76 °C for 5 min and hybridized at 37 °C for 1.5 hr, followed by soaking in 50% formamide for 15 min and washing in 2× SSC twice for 5 min. Specimens were then dehydrated in a series of 75%, 85%, and 100% ethanol baths for 3 min each, incubated with Alexa Fluor 594‐conjugated anti‐human CD45 at room temperature for 1 hr, and washed twice with 0.2% bovine serum albumin. Following the addition of DAPI, slides were observed along the “S” track using a microscope (Nikon). CTCs were identified by their staining pattern of CD45‐/DAPI+/CEP8>2. Theoretically, the size and strength of CEP8 signals in the nucleus of the same specimen should be consistent. Close hybridization signals in single cells were referred to as 1. Small‐scale and weak hybridization signals may have been non‐specific and were thus not considered.

### 2.3 Statistical analysis

Statistical analyses were conducted using the SPSS Statistics 22.0 (SPSS, Chicago, IL). Correlations of CTCs with clinicopathological characteristics were examined using the chi‐square test for categorical variables and one‐way ANOVA for continuous variables. Receiver operating characteristics (ROC) curves were plotted to assess the performance of CTCs in an ampullary cancer screening test. DFS and OS were analyzed by using the Kaplan–Meier method and compared via the log‐rank test. Cox's proportional hazards model was performed to identify factors influencing DFS and OS. Factors significantly associated with DFS and OS on univariate analysis (*p* < 0.05) were subjected to the multivariate models. All statistical tests were two‐sided. *p*‐values less than 0.05 were considered significant.

## 3 RESULTS

### 3.1 Patient characteristics

A total of 86 participants comprising 62 patients with ampullary cancer and 24 healthy controls were investigated in this study; their clinical characteristics and pathological information are shown in Table 1. Twenty‐nine of the patients with ampullary cancer (46.8%) were female and 33 (53.2%) were male; their median age was 62 years (range, 34–78 years). Forty‐three patients (69.4%) were diagnosed with well‐ or moderately differentiated disease, while the remaining 19 (30.6%) were diagnosed with poorly differentiated disease. As for pathological disease stage, 52 patients (83.9%) were stage I–II and 10 (15.2%) were stage III–IV. Twenty‐three patients (37.1%) were classified as T1–2 and 39 (62.9%) as T3–4. Lymphatic invasion was noted in 28 patients (45.2%), and distant metastasis accounted for 4 of the 62 cases (6.5%). Twenty‐six patients (41.9%) had died by the time of analysis. The median follow‐up time was 23.8 months (range 6.0–59.8 months). The median DFS was 19.0 months (range 3.0–59.8), while the median OS was 23.5 months (range 3.7–59.8 months). Forty‐one patients (66.1%) underwent both surgery and chemotherapy, 17 (27.4%) underwent surgery alone, 2 (3.2%) underwent chemotherapy alone, and the remaining 2 (3.2%) received only supportive care.

**Table 1:** Clinical characteristics

Variations	Number	CTC <2	CTC ≥2	CTC <3	CTC ≥3	CTC <5	CTC ≥5
Gender							
Female	29	6	23	11	18	21	8
Male	33	13	20	18	15	25	8
*p*			0.111		0.191		0.764
Age							
< 60	28	7	21	12	16	22	6
≥60	34	12	22	17	17	24	10
*p*			0.382		0.575		0.475
Differentiation							
Well or intermediate	43	13	30	21	22	33	10
Poor	19	6	13	8	11	13	6
*p*			0.916		0.624		0.490
Tumor stage							
I–II	52	18	34	26	26	43	9
III–IV	10	1	9	3	7	3	7
*p*			0.241		0.415		**0.002**
Tumor size							
T1–T2	23	6	17	13	10	20	3
T3–T4	39	13	26	16	23	26	13
*p*			0.550		0.237		0.078
Lymph nodes							
N0	34	12	22	17	17	28	6
N1	28	7	21	12	16	18	10
*p*			0.382		0.575		0.106
Metastasis							
M0	58	19	39	28	30	45	13
M1	4	0	4	1	3	1	3
*p*			0.303		0.701		0.083
CEA							
< 10 ug/ml	50	16	34	23	27	38	12
≥10 ug/ml	12	3	9	6	6	8	4
*p*			0.902		0.803		0.767
Median DFS, months	19.0	22.0	18.0	23.8	16.0	22.0	8.0
*p*			0.211		**0.033**		**0.032**
Median OS, months	23.5	26.9	20.0	29.4	18.0	26.5	13.6
*p*			0.150		**0.039**		**0.029**
Treatment							
Surgery + chemotherapy	41	12	29	18	23	32	9
Surgery	17	7	10	10	7	13	4
Chemotherapy	2	0	2	1	1	2	1
Supportive care	2	0	2	0	2	0	2

*p‐*value <0.05 are indicated as bold.

### 3.2 CTC identification

**Figure 1:**
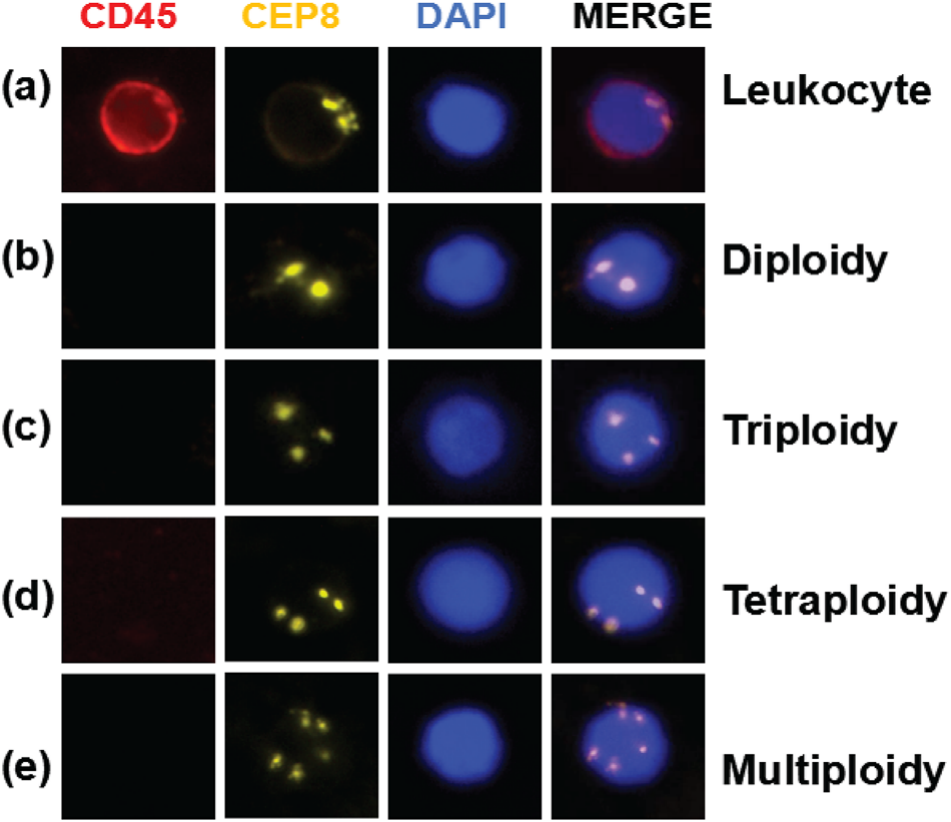
Identification of circulating tumor cells (CTCs). Cells that were CD45‐positive (a) and had ≤2 chromosome 8 centromeres (CEP8) (b) were deemed not to be CTCs. Hyperdiploid cells in peripheral blood samples with no CD45‐positive staining were identified CTCs; shown are a triploid CTC (c), tetraploid CTC (d), and multiploid CTC (e). DAPI, 4′,6‐diamidino‐2‐phenylindole

CD45, DAPI, and CEP8 staining data as obtained under fluorescence microscopy were combined to identify CTCs (Figure 1). Immunostaining for CD45 was performed to discriminate blood cells from tumor cells. Cell nuclei were observed using DAPI, while CEP8 was used to count the chromosome 8 copy number. Cells with CD45 staining (Figure 1a) and/or no more than 2 CEP8 signals (Figure 1b) were not considered CTCs, while those that were CD45‐/DAPI+/CEP>2 were considered to be CTCs. Those with 3 CEP8 signals were classified as triploid CTCs (Figure 1c), those with 4 CEP8 signals tetraploid CTCs (Figure 1d), and those with 5 or more CEP8 signals multiploid CTCs (Figure 1e).

### 3.3 CTCs in patients with ampullary cancer versus control patients

**Figure 2:**
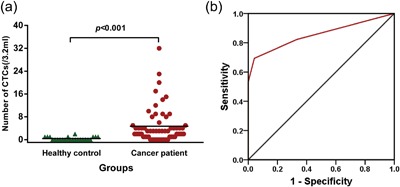
Circulating tumor cells (CTCs) in patients with ampullary cancer and healthy controls. Cells consistent with CTC staining patterns in patients with ampullary cancer were significantly higher than those in healthy controls (*p *< 0.001) (a). Receiver operating characteristic curve for CTC identification by combination staining with CD45, 4′,6‐diamidino‐2‐phenylindole (DAPI), and fluorescence in situ hybridization‐CEP8 in ampullary cancers. The sensitivity of CTCs for the diagnosis of ampullary cancer was 69.4% and the specificity was 95.8% when using a cutoff value of 2 cells/3.2 ml. The area under the curve is 0.854 (95% confidence interval: 0.777–0.931, *p *< 0.001)

The numbers of CTCs were 0–32/3.2 ml (median number, 3 CTCs/3.2 ml) in patients with ampullary cancer and 0–2/3.2 ml (median number, 0 CTCs/3.2 ml) in healthy controls. The difference was statistically significant (*p *< 0.001) (Figure 2a). The presence of ≥1 CTCs/3.2 ml was detected in 51 of 62 patients (82.3%), the presence of ≥2 CTCs/3.2 ml was detected in 43 of 62 patients (69.4%), and the presence of ≥5 CTCs/3.2 ml was detected in 16 of 62 patients (25.8%). Normal tissues can contain small amounts of aneuploid cells, which explains the presence of CD45‐/DAPI+/CEP>2 cells in the peripheral blood of healthy individuals (Biesterfeld, Gerres, Fischer‐Wein, & Bocking, 1994).

We further constructed ROC curves to assess the utility of CTCs as a diagnostic tool for ampullary cancer (Figure 2b). The value of the area under the ROC curve reached 0.854 (95% confidence interval [CI] = 0.777–0.931, *p *< 0.001). Youden's index was then used to select the optimum cutoff value with maximum sensitivity and specificity; this value was 2 CTCs/3.2 ml of whole blood, yielding a sensitivity of 69.4%, and specificity of 95.8%. One out of 24 healthy controls (4.2%) was found to be ‘CTC’‐positive, indicating that our method can yield false positive results.

### 3.4 CTCs and tumor characteristics

We analyzed the relationships between CTCs and clinicopathologic parameters using cutoffs of ≥2, ≥3, and ≥5 CTCs/3.2 ml. Neither ≥2 nor ≥3 CTCs/3.2 ml was associated with most clinicopathologic factors, including sex, age, tumor differentiation, tumor stage, T stage, lymph node invasion, and distant metastasis. However, ≥5 CTCs/3.2 ml was significantly associated with advanced TNM stage (*p* = 0.002) and tended to be associated with higher tumor size (*p* = 0.078), lymph node invasion (*p* = 0.106), and distant metastasis (*p* = 0.083). These results demonstrated that higher CTC levels appear to correlate with a larger tumor burden. CTCs were not associated with elevated carcinoembryonic antigen (CEA) levels. This is consistent with a previous study that found aneuploidy not to be associated with CEA levels (Laubert et al., 2013).

### 3.5 Factors associated with DFS

**Figure 3:**
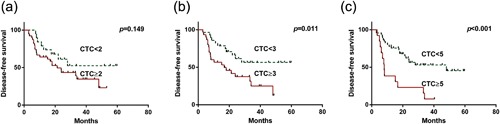
Kaplan–Meier estimates of disease‐free survival according to the number of circulating tumor cells (CTCs) in patients with ampullary cancer. (a) ≥2 versus <2, (b) ≥3 versus <3, and (c) ≥5 versus <5 CTCs/3.2 ml of blood

The relationships between CTCs and DFS are shown in Figure 3. As ≥2 CTCs/3.2 ml did not correlate with shorter DFS (hazard ratio [HR]: 1.8, 95% CI: 0.8–4.0; *p* = 0.156), cutoffs of ≥3 and ≥5 CTCs/3.2 ml were selected to investigate the influence of CTCs on DFS. Median DFS rates were 23.8 and 16.0 months in patients with <3 and ≥3 CTCs/3.2 ml, respectively (*p* = 0.033). The presence of ≥3 CTCs/3.2 ml was associated with poorer DFS on univariate analysis (HR: 2.5, 95% CI: 1.2–5.2, *p* = 0.011; Figure 3 and Table 2). The median DFS rates were 22.0 and 8.0 months in patients with <5 and ≥5 CTCs/3.2 ml, respectively (*p* = 0.032). The presence of ≥5 CTCs/3.2 ml was associated with poorer DFS on univariate analysis (HR: 3.5, 95% CI: 1.7–7.3, *p *< 0.001; Figure 3 and Table 2). Furthermore, poor differentiation, AJCC/Union for International Cancer Control (UICC) T staging, lymph node invasion, and CEA >10 U/ml predicted a significantly poorer DFS rate. All variables that showed a significant correlation on univariate analysis were subjected to Cox regression analysis. Independent predictors of DFS were lymph node invasion (HR: 2.7, 95% CI: 1.3–5.9, *p* = 0.010) and ≥3 CTCs/3.2 ml (HR: 3.0, 95% CI: 1.4–6.4, *p* = 0.004). When ≥5 CTCs/3.2 ml was substituted for ≥3 CTCs/3.2 ml in the multivariate models, ≥5 CTCs/3.2 ml (HR: 3.6, 95% CI: 1.7–7.6, *p* = 0.001) and lymph node invasion (HR: 2.9, 95% CI: 1.4–6.2, *p* = 0.006) remained independent predictors of DFS.

**Table 2:** Factors affecting DFS in patients with ampullary carcinoma

	Univariate		Multivariate for CTC ≥3		Multivariate for CTC ≥5	
	HR (95%CI)	*p* value	HR (95%CI)	*p* value	HR (95%CI)	*p* value
CTC ≥3	2.5 (1.2–5.2)	0.011	3.0 (1.4–6.4)	0.004		
CTC ≥5	3.5 (1.7–7.3)	< 0.001			3.6 (1.7–7.6)	0.001
Sex (male)	0.8 (0.4–1.6)	0.537				
Age (≥60)	1.2 (0.6–2.3)	0.684				
Poor differentiation	2.3 (1.1–4.8)	0.018	‐	‐	‐	‐
AJCC/UICC T staging	2.5 (1.1–5.5)	0.022	‐	‐	‐	‐
Lymph node invasion	2.8 (1.3–5.9)	0.004	2.7 (1.3–5.9)	0.01	2.9 (1.4–6.2)	0.006
Higher TNM staging	2.2 (0.8–5.7)	0.103	‐	‐	‐	‐
CEA >10 U/ml	2.2 (1.0–5.0)	0.048	2.3 (1.0–5.3)	0.055	‐	‐

### 3.6 Factors associated with OS

**Figure 4:**
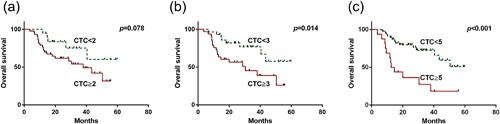
Kaplan–Meier estimates of overall survival according to the number of circulating tumor cells (CTCs) in patients with ampullary cancer. (a) ≥2 versus <2, (b) ≥3 versus <3, and (c) ≥5 versus <5 CTCs/3.2 ml of blood

The median OS rates were 29.4 and 18.0 months in patients with <3 and ≥3 CTCs/3.2 ml, respectively (*p* = 0.039). The presence of ≥3 CTCs/3.2 ml was associated with poorer OS on univariate analysis (HR: 2.7, 95% CI: 1.2–6.3, *p* = 0.014; Figure 4 and Table 3). The median OS rates were 26.5 and 13.6 months in patients with <5 and ≥5 CTCs/3.2 ml, respectively (*p* = 0.029). The presence of ≥5 CTCs/3.2 ml was associated with poorer OS on univariate analysis (HR: 3.8, 95%CI: 1.8–8.5, *p *< 0.001; Figure 3 and Table 3). Demographic features including age and sex were not associated with poorer OS. Tumor‐related factors including poor differentiation, AJCC/UICC T staging, lymph node invasion, distant metastasis, higher TNM staging, and CEA >10 U/ml were significantly associated with poor OS. On multivariate analysis, independent predictors of OS were AJCC/UICC T staging (HR: 3.4, 95% CI: 1.1–10.5, *p* = 0.036), lymph node invasion (HR: 2.6, 95% CI: 1.1–6.3, *p* = 0.034), and distant metastasis (HR: 74.3, 95% CI: 10.2–543.1, *p *< 0.001). The presence of ≥3 CTCs/3.2 ml tended to be an independent predictor of poor OS (HR: 2.5, 95% CI: 1.0–5.4, *p* = 0.055). Additionally, ≥5 CTCs/3.2 ml was an independent predictor of OS on multivariate analysis (HR: 2.8, 95% CI: 1.2–6.5, *p* = 0.020). AJCC/UICC T staging (HR: 3.3, 95% CI: 1.1–10.4, *p* = 0.040), lymph node invasion (HR: 2.7, 95% CI: 1.1–6.8, *p* = 0.027), and distant metastasis (HR: 53.6, 95% CI: 7.2–400.9, *p *< 0.001) were also independent markers of poorer OS.

**Table 3:** Factors affecting OS in patients with ampullary carcinoma

	Univariate		Multivariate for CTC ≥3		Multivariate for CTC ≥5	
	HR (95%CI)	*p* value	HR (95%CI)	*p* value	HR (95%CI)	*p* value
CTC ≥3	2.7 (1.2–6.3)	0.014	2.3 (1.0–5.4)	0.055		
CTC ≥5	3.8 (1.8–8.5)	<0.001			2.8 (1.2–6.5)	0.02
Sex (male)	0.8 (0.4–1.7)	0.559				
Age (≥60)	0.5 (0.2–1.1)	0.069				
Poor differentiation	2.3 (1.1–5.0)	0.029	‐	‐	‐	‐
AJCC/UICC T staging	3.0 (1.1–7.9)	0.021	3.4 (1.1–10.5)	0.036	3.3 (1.1–10.4)	0.04
Lymph node invasion	3.9 (1.7–8.9)	0.001	2.6 (1.1–6.3)	0.034	2.7 (1.1–6.8)	0.027
Distant metastasis	71.8 (12.5–413.7)	<0.001	74.3 (10.2–543.1)	<0.001	53.6 (7.2–400.9)	<0.001
Higher TNM staging	3.9 (1.7–9.1)	0.001	‐	‐	‐	‐
CEA >10 U/ml	2.9 (1.3–6.5)	0.008	‐	‐	‐	‐

## 4 DISCUSSION

In this study, CEP8, CD45, and DAPI were combined to detect CTCs in patients with ampullary cancer and healthy controls. Our method, which used a cutoff of 2 CTCs/3.2 ml whole blood, achieved a sensitivity of 69.4%, and specificity of 95.8%. CTC may serve as a potential prognostic factor for patients with ampullary cancer both in terms of DFS and OS.

The detection rate of CTC depends on the method. The CellSearch system relies on EpCAMs and cytokeratin for cell identification and enumeration, respectively. The CTC detection rates range from 11% to 57% in different tumors types (Allard et al., 2004). As EpCAM and cytokeratin are not expressed in a fair proportion of CTCs, this method has had to be continuously improved. Isolation by size of epithelial tumor cells (ISET) allows highly sensitive enrichment, morphological identification, and enumeration of CTCs (Vona et al., 2000). In one study based on the ISET method, CTCs were found in 18 of 29 patients (62.0%) with primary invasive melanoma and in five out of eight patients (62.5%) with metastatic melanoma (De Giorgi et al., 2010). Khoja et al. (2012) compared CellSearch and ISET in terms of CTC detection in 54 patients with pancreatic cancer, and found that ISET detects a higher number of CTCs than the CellSearch System. Polymerase chain reaction (PCR)‐based methods are also commonly used; CTCs were detected in 33.8–84% of patients by using Nested PCR with *CK20* mRNA as a CTC marker in different studies (Soeth et al., 2005; Zhou et al., 2011). The method of combined immunostaining for CD45, DAPI, and CEP8‐FISH has shown great potential for CTC detection; this method yielded sensitivities of 83.33% and 76.25% for detecting CTCs in lung and ovarian cancers, respectively (Ning et al., 2014). In our study, combined CD45, DAPI, and CEP8‐FISH staining yielded a sensitivity of 69.4% and specificity of 95.8% for ampullary cancer CTC detection. This detection rate was marginally lower than those in lung and ovarian cancers, which may be related to the different biological characteristics of these tumors. To improve the detection rate, immunostaining for cytokeratin was used to detect CTCs; immunostaining of CK, CD45, DAPI, and CEP8/FISH yielded sensitivities and specificities of 68.2% and 94.9% in one pancreatic cancer study (Zhang et al., 2015) and 88% and 90% in another, respectively (Gao et al., 2016). Another technique to improve the detection rate was to capture circulating cells with aneuploidy based on both CEP8 and CEP7 staining. This method yielded a sensitivity of 84% and specificity of 97.6% in patients with lung cancer, which were slightly better than the sensitivity and specificity rates of 76% and 100%, respectively, yielded when using CEP8 alone (Chen & Xu, 2014).

The prognostic significance of CTC has been widely investigated in tumors. A prospective study of 302 patients with nonmetastatic breast cancer showed that ≥1 CTC/7.5 ml was a predictor of poorer PFS and OS (Lucci et al., 2012). A similar result was obtained in a large prospective trial using the CellSearch system in patients with breast cancer (Rack et al., 2014). Studies have also evaluated the prognostic significance of CTC in hepatocellular carcinoma (HCC); a study using the CellSearch system showed that ≥1 CTC per sample was associated with shorter OS on univariate analysis of 59 patients with HCC (*p* = 0.02) (Schulze et al., 2013). A landmark study of CTC using the CellSearch system showed that CTCs were independent predictors of OS in patients with cholangiocarcinoma (Yang et al., 2016). CTCs based on the immunostaining of CD45, DAPI, CK, and FISH‐CEP8 have also been found to be associated with OS; a concentration of ≥3 CTCs/7.5 ml was an independent prognostic factor in patients with pancreatic cancer (Gao et al., 2016). CTCs identified based on FISH‐CEP8 were associated with a curative effect following paclitaxel and cisplatin treatment in patients with advanced gastric cancer (Li et al., 2014). Patients with colon cancer who had CTCs detected by CK20 using real‐time PCR had substantially worse OS rates compared to those without detectable CTCs (Soeth et al., 2005). At the same time, several groups have shown that CTCs are not a prognostic factor for OS in several cancers. There was no statistically significant association between CTCs detected using the CellSearch system and OS in 90 patients with locally advanced rectal cancer who underwent neoadjuvant chemoradiotherapy (Magni et al., 2014). CTCs detected by ISET tended to be associated with lower PFS rates, although without statistical significance (*p* = 0.092) (Oh, Kim, Lee, & Kim, 2017). Few studies have attempted to detect CTCs in patients with ampullary cancer; ours demonstrated that CTCs were detectable in such patients and showed that CTCs are an independent predictor of their survival.

To date, the most reliable prognostic variables in patients with ampullary cancer are lymph node invasion, TNM staging, histologic grade, and serum CEA level (Zhou et al., 2004). Our results are consistent with these previous studies. One study found that patients with intestinal‐type ampullary cancers had better prognoses than those with pancreaticobiliary‐type cancers (Henson, Schwartz, Nsouli, & Albores‐Saavedra, 2009). However, whether the histological type plays a prognostic role is controversial (Zhou et al., 2011). Because the histological types of this cancer have not been well‐defined, we did not explore this aspect. Expression levels of the promyelocytic leukemia tumor suppressor protein and of human equilibrative nucleoside transporter 1, as determined by immunohistochemical staining, were found to be predictive of prognosis in patients with ampullary cancer (Vincenzi et al., 2009). In our study, we showed that CTC levels have good discriminatory power for predicting the prognoses of patients with ampullary cancer as well. As a consequence, CTCs may represent an ideal target for anticancer therapy.

There are several limitations to our study. The power of our statistical analysis was limited by our small sample size. Furthermore, the CTCs detected in our study were circulating aneuploid cells, whereas circulating diploid tumor cells were not evaluated; therefore, the CTC count identified using our method might have been underestimated. Future research should attempt to improve CTC enumeration and detection methods. It is reassuring that only 1 of the 24 healthy control patients had ≥2 CTCs/3.2 ml; furthermore, 11 of the 62 patients had no detectable CTCs. These outcomes indicate that the incidence of false positive cases in our study was low. However, aneuploid cells are widely present in normal tissues and are associated with cell stress, aging, and other factors. Therefore, it is necessary to examine the role of aneuploid cells in the blood of patients with cholangitis, gastrointestinal inflammation, pancreatitis, and benign tumors to elucidate their roles in comparison to actual CTCs. Another limitation was that our findings may not be applicable to all patients with ampullary cancer since ours was a study at a single referral center. Hence, further studies are required for additional validation in an independent validation cohort. As we did not draw blood from patients after treatment, we did not determine the impact of surgery and chemotherapy on CTCs. Overall, the relationship between CTC trends and prognosis requires further study.

## 5 CONSLUSIONS

In summary, our data indicate a potential clinical value for CTCs based on their detection via combination CD45, DAPI, and CEP8‐FISH staining. CTC levels were shown to be associated with tumor extent, and served as prognostic factors in patients with ampullary cancer. The role of CTCs in identifying therapeutic targets and determining follow‐up treatment ought to be further investigated.

## ETHICS APPROVAL AND CONSENT TO PARTICIPATE

This study was conducted with the approval of the Ethics Committee of Fudan University‐Affiliated Huashan Hospital.

## AVAILABILITY OF DATA AND MATERIAL

The datasets analyzed during the current study are available from the corresponding author on reasonable request.

## CONFLICTS OF INTEREST

The authors declare that they have no conflicts of interest.

## References

[jcp26353-bib-0001] Albores‐Saavedra, J. , Schwartz, A. M. , Batich, K. , & Henson, D. E. (2009). Cancers of the ampulla of vater: Demographics, morphology, and survival based on 5,625 cases from the SEER program. Journal of Surgical Oncology, 100(7), 598 –605. 19697352 10.1002/jso.21374

[jcp26353-bib-0002] Allard, W. J. , Matera, J. , Miller, M. C. , Repollet, M. , Connelly, M. C. , Rao, C. , … Terstappen, L. W. (2004). Tumor cells circulate in the peripheral blood of all major carcinomas but not in healthy subjects or patients with nonmalignant diseases. Clinical Cancer Research, 10(20), 6897 –6904. 15501967 10.1158/1078-0432.CCR-04-0378

[jcp26353-bib-0003] Ang, D. C. , Shia, J. , Tang, L. H. , Katabi, N. , & Klimstra, D. S. (2014). The utility of immunohistochemistry in subtyping adenocarcinoma of the ampulla of vater. The American Journal of Surgical Pathology, 38(10), 1371–1379. 24832159 10.1097/PAS.0000000000000230

[jcp26353-bib-0004] Biesterfeld, S. , Gerres, K. , Fischer‐Wein, G. , & Bocking, A. (1994). Polyploidy in non‐neoplastic tissues. Journal of Clinical Pathology, 47(1), 38–42. 8132807 10.1136/jcp.47.1.38 PMC501754

[jcp26353-bib-0005] Chen, Y. Y. , & Xu, G. B. (2014). Effect of circulating tumor cells combined with negative enrichment and CD45‐FISH identification in diagnosis, therapy monitoring, and prognosis of primary lung cancer. Medical Oncology, 31(12), 240. 25361883 10.1007/s12032-014-0240-0

[jcp26353-bib-0006] Cohen, S. J. , Punt, C. J. , Iannotti, N. , Saidman, B. H. , Sabbath, K. D. , Gabrail, N. Y. , … Meropol, N. J. (2008). Relationship of circulating tumor cells to tumor response, progression‐free survival, and overall survival in patients with metastatic colorectal cancer. Journal of Clinical Oncology, 26(19), 3213–3221. 18591556 10.1200/JCO.2007.15.8923

[jcp26353-bib-0007] Cristofanilli, M. (2006). Circulating tumor cells, disease progression, and survival in metastatic breast cancer. Seminars in Oncology, 33(3 Suppl 9), S9 –S14. 10.1053/j.seminoncol.2006.03.016 16797376

[jcp26353-bib-0008] Danielsen, H. E. , Pradhan, M. , & Novelli, M. (2016). Revisiting tumour aneuploidy—The place of ploidy assessment in the molecular era. Nature Reviews Clinical Oncology, 13(5), 291–304. 10.1038/nrclinonc.2015.208 26598944

[jcp26353-bib-0009] De Giorgi, V. , Pinzani, P. , Salvianti, F. , Panelos, J. , Paglierani, M. , Janowska, A. , … Massi, D. (2010). Application of a filtration‐ and isolation‐by‐size technique for the detection of circulating tumor cells in cutaneous melanoma. Journal of Investigative Dermatology, 130(10), 2440 –2447. 20535130 10.1038/jid.2010.141

[jcp26353-bib-0010] Edge, S. B. , & Compton, C. C. (2010). The American Joint Committee on Cancer: The 7th edition of the AJCC cancer staging manual and the future of TNM. Annals of Surgical Oncology, 17(6), 1471 –1474. 20180029 10.1245/s10434-010-0985-4

[jcp26353-bib-0011] Eisenhauer, E. A. , Therasse, P. , Bogaerts, J. , Schwartz, L. H. , Sargent, D. , Ford, R. , … Verweij, J. (2009). New response evaluation criteria in solid tumours: Revised RECIST guideline (version 1.1). European Journal of Cancer, 45(2), 228–247. 19097774 10.1016/j.ejca.2008.10.026

[jcp26353-bib-0012] Fehm, T. , Sagalowsky, A. , Clifford, E. , Beitsch, P. , Saboorian, H. , Euhus, D. , … Uhr, J . (2002). Cytogenetic evidence that circulating epithelial cells in patients with carcinoma are malignant. Clinical Cancer Research, 8(7), 2073–2084. 12114406

[jcp26353-bib-0013] Gao, Y. , Zhu, Y. , Zhang, Z. , Zhang, C. , Huang, X. , & Yuan, Z. (2016). Clinical significance of pancreatic circulating tumor cells using combined negative enrichment and immunostaining‐fluorescence in situ hybridization. Journal of Experimental & Clinical Cancer Research, 35, 66. 27066900 10.1186/s13046-016-0340-0 PMC4828870

[jcp26353-bib-0014] Henson, D. E. , Schwartz, A. M. , Nsouli, H. , & Albores‐Saavedra, J. (2009). Carcinomas of the pancreas, gallbladder, extrahepatic bile ducts, and ampulla of vater share a field for carcinogenesis: A population‐based study. Archives of Pathology & Laboratory Medicine, 133(1), 67–71. 19123739 10.5858/133.1.67

[jcp26353-bib-0015] Khoja, L. , Backen, A. , Sloane, R. , Menasce, L. , Ryder, D. , Krebs, M. , … Dive, C. (2012). A pilot study to explore circulating tumour cells in pancreatic cancer as a novel biomarker. British Journal of Cancer, 106(3), 508–516. 22187035 10.1038/bjc.2011.545 PMC3273340

[jcp26353-bib-0016] Laubert, T. , Bente, V. , Freitag‐Wolf, S. , Voulgaris, H. , Oberlander, M. , Schillo, K. , … Habermann, J. K. (2013). Aneuploidy and elevated CEA indicate an increased risk for metachronous metastasis in colorectal cancer. International Journal of Colorectal Disease, 28(6), 767–775. 23296402 10.1007/s00384-012-1625-1

[jcp26353-bib-0017] Li, Y. , Zhang, X. , Ge, S. , Gao, J. , Gong, J. , Lu, M. , … Shen, L. (2014). Clinical significance of phenotyping and karyotyping of circulating tumor cells in patients with advanced gastric cancer. Oncotarget, 5(16), 6594–6602. 25026283 10.18632/oncotarget.2175 PMC4196148

[jcp26353-bib-0018] Lucci, A. , Hall, C. S. , Lodhi, A. K. , Bhattacharyya, A. , Anderson, A. E. , Xiao, L. , … Krishnamurthy, S. (2012). Circulating tumour cells in non‐metastatic breast cancer: A prospective study. Lancet Oncology, 13(7), 688–695. 22677156 10.1016/S1470-2045(12)70209-7

[jcp26353-bib-0019] Magni, E. , Botteri, E. , Ravenda, P. S. , Cassatella, M. C. , Bertani, E. , Chiappa, A. , … Zampino, M. G. (2014). Detection of circulating tumor cells in patients with locally advanced rectal cancer undergoing neoadjuvant therapy followed by curative surgery. International Journal of Colorectal Disease, 29(9), 1053 –1059. 25008360 10.1007/s00384-014-1958-z

[jcp26353-bib-0020] Nagrath, S. , Jack, R. M. , Sahai, V. , & Simeone, D. M. (2016). Opportunities and challenges for pancreatic circulating tumor cells. Gastroenterology, 151(3), 412–426. 27339829 10.1053/j.gastro.2016.05.052

[jcp26353-bib-0021] Ning, N. , Zhan, T. , Zhang, Y. , Chen, Q. , Feng, F. , Yang, Z. , … Cui, W. (2014). Improvement of specific detection of circulating tumor cells using combined CD45 staining and fluorescence in situ hybridization. Clinica Chimica Acta, 433, 69–75. 10.1016/j.cca.2014.02.019 24607330

[jcp26353-bib-0022] Oh, B. Y. , Kim, J. , Lee, W. Y. , & Kim, H. C. (2017). A new size‐based platform for circulating tumor cell detection in colorectal cancer patients. Clinical Colorectal Cancer, 16(3), 214–219. 28209483 10.1016/j.clcc.2017.01.007

[jcp26353-bib-0023] Pecot, C. V. , Bischoff, F. Z. , Mayer, J. A. , Wong, K. L. , Pham, T. , Bottsford‐Miller, J. , … Sood, A. K. (2011). A novel platform for detection of CK+ and CK‐ CTCs. Cancer Discovery, 1(7), 580–586. 22180853 10.1158/2159-8290.CD-11-0215 PMC3237635

[jcp26353-bib-0024] Plaks, V. , Koopman, C. D. , & Werb, Z. (2013). Cancer: Circulating tumor cells. Science, 341(6151), 1186–1188. 24031008 10.1126/science.1235226 PMC3842225

[jcp26353-bib-0025] Rack, B. , Schindlbeck, C. , Juckstock, J. , Andergassen, U. , Hepp, P. , Zwingers, T. , … SUCCESS Study Group. (2014). Circulating tumor cells predict survival in early average‐to‐high risk breast cancer patients. Journal of the National Cancer Institute, 106(5), dju066 https://doi.org/:10.1093/jnci/dju066 10.1093/jnci/dju066 PMC4112925 24832787

[jcp26353-bib-0026] Schulze, K. , Gasch, C. , Staufer, K. , Nashan, B. , Lohse, A. W. , Pantel, K. , … Wege, H. (2013). Presence of EpCAM‐positive circulating tumor cells as biomarker for systemic disease strongly correlates to survival in patients with hepatocellular carcinoma. International Journal of Cancer, 133(9), 2165 –2171. 23616258 10.1002/ijc.28230

[jcp26353-bib-0027] Siegel, R. L. , Miller, K. D. , & Jemal, A. (2017). Cancer statistics, 2017. CA: A Cancer Journal for Clinicians, 67(1), 7 –30. 28055103 10.3322/caac.21387

[jcp26353-bib-0028] Soeth, E. , Grigoleit, U. , Moellmann, B. , Roder, C. , Schniewind, B. , Kremer, B. , … Vogel, I. (2005). Detection of tumor cell dissemination in pancreatic ductal carcinoma patients by CK 20 RT‐PCR indicates poor survival. Journal of Cancer Research and Clinical Oncology, 131(10), 669 –676. 16136352 10.1007/s00432-005-0008-1 PMC12161178

[jcp26353-bib-0029] Sonpavde, G. , & Antonarakis, E. S. (2017). Circulating tumor cells in advanced prostate cancer: Time to move from prognostic to predictive ability. European Urology, 71(2), 172 –173. 27591932 10.1016/j.eururo.2016.08.053

[jcp26353-bib-0030] Vincenzi, B. , Santini, D. , Perrone, G. , Russo, A. , Adamo, V. , Rizzo, S. , … Tonini, G. (2009). Promyelocytic leukemia (PML) gene expression is a prognostic factor in ampullary cancer patients. Annals of Oncology, 20(1), 78–83. 18689862 10.1093/annonc/mdn558

[jcp26353-bib-0031] Vona, G. , Sabile, A. , Louha, M. , Sitruk, V. , Romana, S. , Schutze, K. , … Paterlini‐Bréchot, P. (2000). Isolation by size of epithelial tumor cells: a new method for the immunomorphological and molecular characterization of circulatingtumor cells. The American Journal of Pathology, 156(1), 57 –63. 10623654 10.1016/S0002-9440(10)64706-2 PMC1868645

[jcp26353-bib-0032] Yang, J. D. , Campion, M. B. , Liu, M. C. , Chaiteerakij, R. , Giama, N. H. , Ahmed, M. H. , … Roberts, L. R. (2016). Circulating tumor cells are associated with poor overall survival in patients with cholangiocarcinoma. Hepatology, 63(1), 148 –158. 26096702 10.1002/hep.27944 PMC4684812

[jcp26353-bib-0033] Zhang, Y. , Wang, F. , Ning, N. , Chen, Q. , Yang, Z. , Guo, Y. , … Cui, W. (2015). Patterns of circulating tumor cells identified by CEP8, CK, and CD45 in pancreatic cancer. International Journal of Cancer, 136(5), 1228–1233. 25042121 10.1002/ijc.29070

[jcp26353-bib-0034] Zhou, H. , Schaefer, N. , Wolff, M. , & Fischer, H. P. (2004). Carcinoma of the ampulla of Vater: Comparative histologic/immunohistochemical classification and follow‐up. The American Journal of Surgical Pathology, 28(7), 875 –882. 15223956 10.1097/00000478-200407000-00005

[jcp26353-bib-0035] Zhou, J. , Hu, L. , Yu, Z. , Zheng, J. , Yang, D. , Bouvet, M. , & Hoffman, R. M. (2011). Marker expression in circulating cancer cells of pancreatic cancer patients. Journal of Surgical Research, 171(2), 631–636. 20869080 10.1016/j.jss.2010.05.007

